# Ferulic acid ameliorates TNBS-induced ulcerative colitis through modulation of cytokines, oxidative stress, iNOs, COX-2, and apoptosis in laboratory rats

**DOI:** 10.17179/excli2016-393

**Published:** 2016-08-09

**Authors:** Smeeta S. Sadar, Niraj S. Vyawahare, Subhash L. Bodhankar

**Affiliations:** 1 Padmashree Dr. D. Y. Patil College of Pharmacy, Akurdi, Pune Maharashtra, 411044, India; 2 Department of Pharmacology, Poona College of Pharmacy, Bharati Vidyapeeth Deemed University, Pune, Maharashtra, 411038, India

**Keywords:** ferulic acid, TNBS-induced colitis, apoptosis, TNF-alpha, IL-1beta, IL-6, COX-2, iNOs

## Abstract

Ulcerative colitis (UC) is a chronic immune-inflammatory disorder characterized by oxido-nitrosative stress, the release of pro-inflammatory cytokines and apoptosis. Ferulic acid (FA), a phenolic compound is considered to possess potent antioxidant, anti-apoptotic and anti-inflammatory activities. The aim is to evaluate possible mechanism of action of FA against trinitrobenzensulfonic acid (TNBS) induced ulcerative colitis (UC) in rats. UC was induced in Sprague-Dawley rats (150-200 g) by intrarectal administration of TNBS (100 mg/kg). FA was administered (10, 20 and 40 mg/kg, p.o.) for 14 days after colitis was induced. Various biochemical, molecular and histological changes were assessed in the colon. Intrarectal administration of TNBS caused significant induction of ulcer in the colon with an elevation of oxido-nitrosative stress, myeloperoxidase and hydroxyproline activity in the colon. Administration of FA (20 and 40 mg/kg) significantly decrease oxido-nitrosative stress, myeloperoxidase, and hydroxyproline activities. Up-regulated mRNA expression of TNF-α, IL-1β, IL-6, COX-2, and iNOs, as well as down-regulated IL-10 mRNA expressions after TNBS administration, were significantly inhibited by FA (20 and 40 mg/kg) treatment. Flow cytometric analysis revealed that intrarectal administration of TNBS-induced significantly enhanced the colonic apoptosis whereas administration of FA (20 and 40 mg/kg) significantly restored the elevated apoptosis. FA administration also significantly restored the histopathological aberration induced by TNBS. The findings of the present study demonstrated that FA ameliorates TNBS-induced colitis via inhibition of oxido-nitrosative stress, apoptosis, proinflammatory *cytokines production, and down- regulation of COX-2 synthesis.*

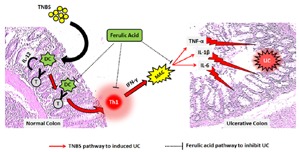

**Graphical Abstract:** TNBS caused activation of T cells which interact with CD40 on antigen presenting cells i.e. dendritic cells (DC) that induce the key Interleukin 12 (IL-12)-mediated Th1 T cell immune inflammatory response. It releases interferon-γ (IFN-γ), which in turn induces macrophages (MAC) to produce TNF-α and other pro-inflammatory cytokines (e.g., IL-1β, IL-6). This inflammatory influx resulted in induction of ulcerative colitis (UC). Administration of FA may inhibit this IFN-γ induced inflammatory cascade via a decrease in the release of pro-inflammatory cytokines to ameliorate TNBS-induced colitis.

## Abbreviations

Cyclooxygenase-2 (COX-2), Dimethyl sulfoxide (DMSO), Glutathione (GSH), Inducible nitric oxide synthase (iNOs), Inflammatory Bowel Disease (IBD), Interleukin's (IL's), Malondialdehyde (MDA), Myeloperoxidase (MPO), Reactive Oxygen Species (ROS), Sulfasalazine (SLZ), Superoxide Dismutase (SOD), Trinitrobenzene Sulfonic Acid (TNBS), Tumour necrosis factor-α (TNF-α)

## Introduction

Inflammatory bowel disease (IBD) is a chronic, relapsing, idiopathic clinicopathological condition where mucosal inflammation and hemorrhage lead to colonic damage (Andres and Friedman, 1999[2]). Diarrhea and abdominal pain are the clinically discernible symptom of patients suffering from IBD. Ulcerative colitis (UC) and Crohn's disease (CD) are two debilitating and chronic disorders of gastrointestinal system collectively referred as IBD. With increased industrialization, the incidence and prevalence of IBD have surged worldwide affecting up to 0.5 % of the general population. It has been reported that incidence of IBD has increased over a few decades ranging from 10 to 30 per 100,000 (Kaplan, 2015[39]). Higher risk of colon cancer is associated with IBD patients besides significantly decreased the quality of life. 

It has been well documented that an array of etiopathogenic factors including genetic modulation, infective agents, immunological disturbance, and smoking are the major risk factor for the development of IBD (Kandhare et al., 2016[31]). Furthermore, altered pro-inflammatory cytokines (tumour necrosis factor-α (TNF-α), interleukin (IL)), anti-inflammatory cytokines (IL-4 and IL-10), glycosaminoglycan content in gastric mucosa, increased oxidative stress and intestinal permeability are the important hallmark of IBD (Kandhare et al., 2012[35]). Evidence from the previous studies suggested that gastrointestinal (GI) tract is more susceptible to damage induced by oxidants (Kandhare et al., 2013[30]; Kumar et al., 2014[45]). It may result from repetitive exposure of gastric mucosa to various stresses that increased interaction of immune cells, dietary factors, and intestinal flora in the GI tract. 

An array of animal models of experimental colitis has been employed to evaluate the potential of various therapeutic moieties against IBD. Among all animal models of IBD, 2,4,6- trinitrobenzene sulfonic acid (TNBS)-induced colitis is well established, easily induced and highly reproducible model that mimics most of the clinicopathological symptoms of ulcerative colitis (de Faria et al., 2012[14]; Kandhare et al., 2011[36]; Randhawa et al., 2014[54]). When TNBS is delivered intra-rectally, the immunological inflammation results from a formation of covalent binding of haptenates autologous colonic proteins with a trinitrophenyl moiety which induces an IL-12-mediated Th1 T cell transmural colitis, a delayed-type hypersensitivity (Hoffmann et al., 2002[23]). This IL-12-mediated Th1 T cell transmural colitis has resemblance with human IBD in many features including histological and immunological alterations (Randhawa et al., 2014[54]).

The current clinical practice guidelines indicate that IBD should be treated using 5-aminosalicylic acid (5-ASA), sulfasulfapyridine (SASP), broad spectrum antibiotics, steroids, and immunosuppressant. These interventions inhibit the inflammatory mediators via various mechanisms leading to downregulation of the immune and inflammatory responses of IBD (Kandhare et al., 2013[30]). However, these treatment strategies are associated with adverse effects (such as nausea and vomiting, skin rashes, hepatitis, hematologic abnormalities, folate deficiency, pancreatitis, systemic lupus erythematosus, etc.) and high relapse rate that limit their clinical usage (Joshi et al., 2005[28]). Hence, there is an inevitable need to develop a therapeutic strategy which overcomes these adverse events. Herbal drugs provide a ray of hope in the development of novel and therapeutically acceptable drugs with limited adverse reaction profile. Few systematic reviews of randomized control trials have found herbal drugs as a promising therapy for the treatment of IBD (Ng et al., 2013[51]; Triantafyllidi et al., 2015[60]). Moreover, most of the published trials showed no side effects. In fact, the number and type of side effects were similar to those of placebo or mesalazine. It is quite important in patients with previous operations or patients who experienced significant side effects being on conventional treatment. Hence, larger controlled studies with stricter endpoints and better-defined patient groups are required to obtain more conclusive results on the use of Complementary and Alternative Medicine (CAM) therapies in IBD. In the light of the experimental evidence, it is assumed that novel herbal moieties are explored as possible drug candidates/pharmacophores.

Polyphenols are chemical moieties present abundantly in various natural compounds. These compounds are well-established antioxidants with excellent free radical quenching ability, and play an important role in amelioration of various immune-inflammatory diseases which including arthritis, allergic rhinitis, asthma and IBD (Vauzour et al., 2010[61]). Ferulic acid (4-hydroxy-3-methoxycinnamic acid), a polyphenolic compound present abundantly in various natural plants including rice, wheat, oats, pineapple, grasses, grains, vegetables, flowers, fruits, leaves, beans, seeds of coffee, artichoke, peanut and nuts (Kumar and Pruthi, 2014[43]). Moreover, cell walls of cereal grains and a variety of food plants (pineapple, bananas, spinach, and beetroot) contain extractable amount (0.5-2 %) of FA, and highest known concentration of ferulic acid (FA) glucoside has been found in flax seed (4.1 ± 0.2 g/kg) (Besseau et al., 2007[9]; Kumar and Pruthi, 2014[43]). FA possesses a plethora of pharmacological properties such as antioxidant, free radicals scavenger, anticancer, antihyperlipidemic, antihypertensive, antidiabetic, antimicrobial, radioprotective, cardioprotective, neuroprotective, anti-carcinogenic and hepatoprotective potential (Baskaran et al., 2010[6]; Bourne and Rice-Evans, 1998[12]). In addition, FA inhibits the expression of cytotoxic enzymes such as cyclooxygenase-2 (Ojha et al., 2015[53]). The resonance stabilization ability of FA is mainly responsible for its antioxidant and anti-inflammatory activity in carrageenan-induced rat paw edema model (Chawla et al., 1987[13]). Earlier studies have suggested that FA protects brain cells through inhibition of hydroxyl and peroxyl radicals (Kanski et al., 2002[38]; Ogiwara et al., 2001[52]). FA has the ability to promote healing via inhibition of lipid peroxidation and nitric oxide synthase (Ghaisas et al., 2014[18]). The previous study has shown that Brazilian Green Propolis possesses antiulcer potential mainly due to the presence of FA in it (Barros et al., 2008[5]). However, its therapeutic activity against TNBS-induced IBD has not been experimentally evaluated. Thus, the aim of present investigation was to evaluate the potential of FA against TNBS-induced experimental colitis in laboratory animals by investigating various biochemical, molecular and histological changes.

## Material and Methods

### Animals

Adult Sprague Dawley rats (150-200 g) were obtained from the National Institute of Biosciences, Pune, India. They were maintained at 24 ± 1° C, with a relative humidity of 45-55 % and 12:12 h dark/light cycle. The animals had free access to standard pellet chow (Pranav Agro Industries Ltd., Sangli, India) and water throughout the experimental protocol. All experiments were carried out between 09:00 and 17:00 h. The experimental protocol was approved by the Institutional Animal Ethics Committee (IAEC) of Poona College of Pharmacy, Pune and performed in accordance with the guidelines of Committee for Control and Supervision of Experimentation on Animals (CPCSEA).

### Drugs and chemicals

Ferulic acid and TNBS were purchased from Sigma Chemical Co. (St Louis, MO, USA). Sulfasalazine was obtained as a gift sample from Symed Pharmaceutical Pvt. Ltd., Hyderabad. Total RNA Extraction kit and One-step Reverse transcription-polymerase chain reaction (RT-PCR) kit was purchased from MP Biomedicals India Private Limited, India.

### Induction of colitis and drug treatment schedule

Colitis was induced according to the procedure described elsewhere (Li et al., 2011[47]). Briefly, rats were slightly anaesthetized with ether following a 24 h fast, and then TNBS dissolved in 50 % ethanol was instilled into the colon of the animals (100 mg in a volume of 0.25 mL) using a medical-grade polyurethane catheter for enteral feeding (external diameter 2 mm) inserted 8 cm into the anus. Different control groups were created for comparison with TNBS/ethanol instillation: rats in the sham, as well as ethanol, treated group received an enema of physiological saline instead of the TNBS solution, and ethanol treated group received 0.25 mL of 50 % ethanol. Rats were randomly divided into following groups of 10-12 rats as follows:

1. Group I Sham Control: Received physiological saline in a volume of 0.25 mL (once, intra-rectally) and 1 ml of 1 % aqueous solution of Dimethyl sulfoxide (DMSO) for 14 days
2. Group II Ethanol treated animals: Received ethanol (50 %) in a volume of 0.25 mL (once, intra-rectally) and 1 ml of 1 % aqueous solution of DMSO for 14 days
3. Group III TNBS-induced control animals: Received TNBS (100 mg/kg) in a volume of 0.25 mL (once, intra-rectally) and 1 ml of 1 % aqueous solution of DMSO for 14 days
4. Group IV TNBS-induced and Sulfasalazine (50 mg/kg) treated animals: Received TNBS (100 mg/kg) in a volume of 0.25 mL (once, intra-rectally) and treatment with sulfasalazine (50 mg/kg, p.o.) 14 day
5. Group V TNBS-induced and Ferulic acid (10 mg/kg) treated animals: Received TNBS (100 mg/kg) in a volume of 0.25 mL (once, intra-rectally) and treatment with FA (10 mg/kg, p.o.) 14 day
6. Group VI TNBS-induced and Ferulic acid (20 mg/kg) treated animals: Received TNBS (100 mg/kg) in a volume of 0.25 mL (once, intra-rectally) and treatment with FA (20 mg/kg, p.o.) 14 day
7. Group VII TNBS-induced and Ferulic acid (40 mg/kg) treated animals: Received TNBS (100 mg/kg) in a volume of 0.25 mL (once, intra-rectally) and treatment with FA (40 mg/kg, p.o.) 14 day

The details of the experimental paradigm are presented in the Supplementary File 1. The selection of dose for FA was based on the studies carried out by the previous researcher (Balasubashini et al., 2004[4]). FA (1 % aqueous solution of DMSO) was administered to rats in the three different dosages (10, 20 and 40 mg/kg, p.o., once a day) for 14 consecutive days. Sulfasalazine (1 % aqueous solution of DMSO) was given in a dose of 50 mg/kg/day orally in rats (Hyam et al., 2013[26]). On the 15^th^ day, rats were sequentially anesthetized with anesthetic ether for about 30-40 s. The blood was withdrawn by retro-orbital puncture. Each blood sample was collected into separate vials for determination of serum parameters. After blood collection, the animals were sacrificed by cervical dislocation; the colon was excised then assessment of colonic damage was performed and frozen immediately in liquid nitrogen and stored at -80° C for further biochemical (n = 5) and molecular examination (n = 4). A part of the freshly excised colon of three animals from each group was washed with saline and preserved in 10 % formaldehyde solution for histopathological studies.

### Assessment of colonic damage, ulcer area and ulcer index

The severity of colitis was evaluated by an independent observer who was blinded to the treatment. For each animal, the distal 10 cm portion of the colon was removed and cut longitudinally, slightly cleaned in physiological saline to remove fecal residues and weighed. Macroscopic inflammation scores were assigned based on clinical features of the colon. The presence of adhesions (score 0-10) and stool consistency (score 0-4) were evaluated according to the previously reported methods (Kandhare et al., 2013[30]; Kumar et al., 2014[45]). Ulcer area and ulcer index were assessed according to previously reported methods (Kandhare et al., 2013[30]; Kumar et al., 2014[45]).

### Biochemical assays

For colon homogenization, tissue segments were mixed with 0.1 M phosphate buffer and homogenized on ice for 60 sec at 10000 r.p.m. in a homogenizer (Remi Equipment Pvt. Ltd., Remi Motors Ltd., Mumbai, India). Supernatant of tissue homogenates was employed to estimate superoxide dismutase (SOD), reduced glutathione (GSH), lipid peroxidation (MDA activity) and nitric oxide (NO activity) as described previously (Kandhare et al., 2013[30]; Kumar et al., 2014[45]). The colonic myeloperoxidase assay and hydroxyproline activity were determined according to the previously described method (Kandhare et al., 2013[30]; Kumar et al., 2014[45]).

### RNA extraction and RT-PCR analysis

The levels of mRNA were analyzed in colon tissue using RT-PCR. Single-stranded cDNA was synthesized from 5 μg of total cellular RNA using reverse transcriptase kit (MP Biomedicals India Private Limited, India) as described previously (Kandhare et al., 2012[33]). The primer sequence for tumor necrosis factor-α (TNF-α), Interleukin-1β (IL-1β), Interleukin-6 (IL-6), Interleukin-10 (IL-10), Cyclooxygenase-2 (COX-2), inducible nitric oxide synthase (iNOs), and β-actin are presented in the Supplementary File 2. Amplification of β-actin served as a control for sample loading and integrity. PCR products were detected by electrophoresis on a 1.5 % agarose gel containing ethidium bromide. The size of amplicons was confirmed using a 100-bp ladder as a standard size marker. The amplicons were visualized, and images were captured using a gel documentation system (Alpha Innotech Inc., San Leandro, CA, USA). Expression of all the genes was assessed by generating densitometry data for band intensities in different sets of experiments and was generated by analyzing the gel images on the Image J software (Version 1.33, Wayne Rasband, NIH, Bethesda, MD, USA) semi-quantitatively. The band intensities were compared with constitutively expressed β-actin. The intensity of mRNAs was standardized against that of the β-actin mRNA from each sample, and the results were expressed as PCR-product/ β-actin mRNA ratio.

### Flow cytometry analysis

Preparation of lamina propria mononuclear cell (LPMC) suspensions and determination of apoptotic cell populations were determined as previously described (Kandhare et al., 2012[33]). At the end of treatment, the colon of rats was collected and mixed with 0.4 % collagenase and 0.25 % Trypsin at 37° C for 30 min and dissociated, grinded and obtained homogenate was passed through a 70 μm nylon mesh. LPMC suspension was washed three times with phosphate-buffered saline (PBS). In order to determine LPMC apoptosis, the isolated LPMCs were incubated with rabbit anti-cow S-100 antibody and followed by staining with APC-goat anti-rabbit IgG (both from BD) with FITC-Annexin V and PI (Sigma-Aldrich, St. Louis, MO, USA). The percentages of expression of Fas and Annexin-V on gated S-100 positive LPMC were analyzed by a Fluorescence-activated cell sorting (FACS) Calibur cytometer using CELL Quest software (Becton & Dickinson, San Diego, USA).

### Evaluation based on microscopic (histologic) characters

Freshly excised colon of three animals from each group was washed with saline and preserved in 10 % formaldehyde solution for histopathological studies. The specimens were dehydrated and placed in xylene for 1 hour (3 times) and later in ethyl alcohol (70 %, 90 %, and 100 % respectively) for 2 hours. Then paraffin-embedded tissue sections cut at five μm thickness were prepared and stained after deparaffination using hematoxylin and eosin stain (H & E). Sections were examined under a light microscope (an optical microscope with Nikon E200 camera) to obtain a general impression of the histopathology features of a specimen such as thickening of the mucosa, destruction of mucosal epithelium, inflammatory cell infiltration, submucosal edema, necrosis and ulceration (Kandhare et al., 2013[30]). Photomicrographs were captured at a magnification of 40 X and 100 X. 

### Statistical analysis

All the results were expressed as mean ± S.E.M. Data analysis was performed using GraphPad Prism 5.0 software (GraphPad, San Diego, CA). Statistical comparisons were made between drug-treated groups and TNBS control animals. Data analyzed using one-way ANOVA followed by Tukey's multiple range test. Data of macroscopical score and stool consistency score was analyzed using nonparametric Kruskal-Wallis ANOVA. A value of *P < *0.05 was considered to be statistically significant.

## Results

### Effect of FA on TNBS-induced alteration in body weight, colon weight to length ratio, ulcer area, ulcer index, macroscopic scores and stool consistency

There was significant decrease (*p* < 0.05) in the body weight whereas colon weight to length ratio, macroscopic scores, and stool consistency score were significantly increased (*p* < 0.05) in TNBS-induced control rats as compared to sham as well as ethanol treated rats. Intra-rectal instillation of TNBS significantly increased (*p* < 0.05) the ulcer area and ulcer index in TNBS-induced control rats as compared to sham as well as ethanol treated rats. When compared with TNBS-induced control rats, treatment with FA (20 and 40 mg/kg) showed significant inhibition (*p* < 0.05) in TNBS-induced alterations in body weight, colon weight to length ratio, macroscopic scores, and stool consistency. However, FA (20 and 40 mg/kg) treatment significantly decreased (*p* < 0.05) ulcer area and index compared to TNBS-induced control rats. Treatment with sulfasalazine (350 mg/kg) also significantly increased (*p* < 0.05) body weight and significantly decreased (*p* < 0.05) colon weight to length ratio, macroscopic scores and stool consistency score as compared to TNBS-induced control rats. When compared with TNBS-induced control rats, sulfasalazine (350 mg/kg) treated rats also showed significant amelioration (*p* < 0.05) of increased ulcer area and ulcer index. Moreover, increased body weight as well as decreased in colon weight to length ratio, macroscopic scores, and stool consistency score was more significant (*p* < 0.05) in sulfasalazine (350 mg/kg) treated rats as compared to FA (10 and 20 mg/kg) treated rats. When compared with FA (20 mg/kg) treatment, FA (40 mg/ kg) significantly (*p* < 0.05) restore the altered ulcer area, ulcer index, macroscopic scores, and stool consistency (Table 1(Tab. 1)).

### Effect of FA on TNBS-induced alteration in oxido-nitrosative stress

The colonic SOD and GSH levels were significantly decreased (*p* < 0.05) while colonic MDA and NO levels were significantly increased (*p* < 0.05) in TNBS-induced control rats after intrarectal administration of TNBS as compared to sham as well as ethanol treated rats. Administration of FA (10 mg/kg) failed to show any significant changes in increased oxido-nitrosative stress as compared to TNBS-induced control rats. However, FA (20 and 40 mg/kg) treatment significantly increased (*p* < 0.05) the levels of colonic SOD and GSH; whereas significantly decreased (*p* < 0.05) the colonic MDA and NO levels as compared to TNBS-induced control rats. When compared with TNBS-induced control rats, sulfasalazine (350 mg/kg) treatment also significantly restored (*p* < 0.05) in the TNBS-induced alterations in oxido-nitrosative stress. Moreover, sulfasalazine administration significantly decreased (*p *< 0.05) the colonic oxido-nitrosative stress as compared to FA (20 and 40 mg/kg) treated rats (Table 2(Tab. 2)).

### Effect of FA on TNBS-induced alteration in colonic MPO and hydroxyproline activity

There was a significant increase (*p* < 0.05) in colonic MPO and hydroxyproline activity in TNBS-induced control rats as compared to sham as well as ethanol treated rats. FA (20 and 40 mg/kg) treatment significantly decreased (*p* < 0.05) the colonic MPO and hydroxyproline activity as compared to TNBS-induced control rats. However, there was non-significant decreased in colonic MPO and hydroxyproline activity in FA (20 and 40 mg/kg) treated rats as compared to TNBS-induced control rats. When compared with TNBS-induced control rats, sulfasalazine (350 mg/kg) treatment also showed the significant decrease (*p* < 0.05) in colonic MPO and hydroxyproline activity (Table 2(Tab. 2)).

### Effect of FA on TNBS-induced alteration in colonic TNF-α, IL-1β, IL-6, and IL-10 mRNA expressions

**Figure 1:**
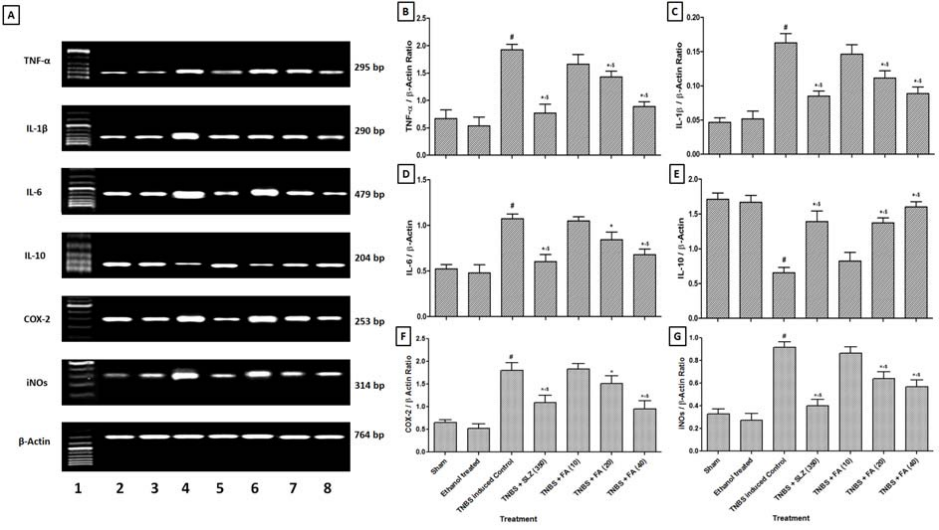
Effect of FA on TNBS-induced alteration in colonic TNF-α, IL-1β, IL-6, IL-10, COX-2 and iNOs mRNA expression in rats (A), quantitative representation of mRNA expression of TNF-α (B), IL-1β (C), IL-6 (D), IL-10 (E), COX-2 (F) and iNOs (G). Data are expressed as mean ± SEM (n = 4) and analyzed by one-way ANOVA followed by Tukey's multiple range test. *p < 0.05 as compared to the TNBS-induced control group, ^#^p < 0.05 as compared to sham as well as ethanol-treated control group and ^$^p < 0.05 as compared to one another. SLZ (350): TNBS-induced and Sulfasalazine (350 mg/kg, p.o.) treated group; FA (10): TNBS-induced and Ferulic acid (10 mg/kg, p.o.) treated group; FA (20): TNBS-induced and Ferulic acid (20 mg/kg, p.o.) treated group and FA (40): TNBS-induced and Ferulic acid (40 mg/kg, p.o.) treated group. Lane 1: Ladder 1000 bp, Lane 2: mRNA expression of sham group, Lane 3: mRNA expression of ethanol-treated control group, Lane 4: mRNA expression of TNBS-induced control group, Lane 5: mRNA expression of TNBS-induced and sulfasalazine (350 mg/kg, p.o.) treated group, Lane 6: mRNA expression of TNBS-induced and FA (10 mg/kg, p.o.) treated group, Lane 7: mRNA expression of TNBS-induced and FA (20 mg/kg, p.o.) treated group and Lane 8: mRNA expression of TNBS-induced and FA (40 mg/kg, p.o.) treated group.

Intra-rectal instillation of TNBS significantly upregulated (*p* < 0.05) colonic TNF-α, IL-1β, and IL-6, mRNA expression and colonic IL-10 mRNA expression was down-regulated significantly (*p* < 0.05) in TNBS-induced control rats as compared to sham as well as ethanol treated rats. Upregulated TNF-α, IL-1β and IL-6 mRNA expressions were significantly down-regulated (*p* < 0.05) by FA (20 and 40 mg/kg) treatment whereas IL-10 mRNA expression was significantly upregulated (*p* < 0.05) by FA (20 and 40 mg/ kg) treatment as compared to TNBS-induced control rats. On the other hand, FA (10 mg/ kg) treatment failed to show a significant effect on TNBS-induced changes on colonic mRNA expressions of TNF-α, IL-β, and IL-6 levels. Furthermore, sulfasalazine (350 mg/ kg) treatment showed the significantly down-regulated (*p* < 0.05) colonic TNF-α, IL-1β, and IL-6 mRNA expression while significantly up-regulated (*p* < 0.05) colonic IL-10 mRNA expression as compared to TNBS-induced control rats. Moreover, up-regulation of colonic IL-10 mRNA expression by FA (40 mg/kg) treatment was significant (*p* < 0.05) as compared to sulfasalazine (350 mg/kg) treatment. Furthermore, treatment with FA (40 mg/kg) showed significant (*p* < 0.05) down-regulation in TNF-α and IL-1β mRNA expression as compared to FA (20 mg/kg) treatment (Figure 1(Fig. 1)).

### Effect of FA on TNBS-induced alteration in colonic COX-2 and iNOs mRNA expressions

There was significant upregulation (*p* < 0.05) in colonic COX-2 and iNOs mRNA expressions in TNBS-induced control rats after intrarectal administration of TNBS as compared to sham as well as ethanol treated rats. FA (20 and 40 mg/kg) treatment significantly down-regulated (*p* < 0.05) colonic COX-2 and iNOs mRNA expressions as compared to TNBS-induced control rats. However, FA (10 mg/kg) treatment failed to show any significant down-regulation in colonic COX-2 and iNOs mRNA expressions as compared to TNBS control rat. When compared with TNBS control rats, sulfasalazine (350 mg/kg) treatment also showed the significant down-regulation (*p* < 0.05) of colonic COX-2 and iNOs mRNA expression. Whereas, this down-regulation in the colonic COX-2 mRNA expression was more significant (*p* < 0.05) in the FA (40 mg/kg) treated rats than sulfasalazine (350 mg/kg) treated rats. FA (40 mg/kg) treatment showed significant (*p* < 0.05) down-regulation in colonic COX-2 mRNA expressions as compared to FA (20 mg/kg) treatment (Figure 1(Fig. 1)).

### Effect of FA on TNBS-induced alteration in colonic apoptosis

**Figure 2:**
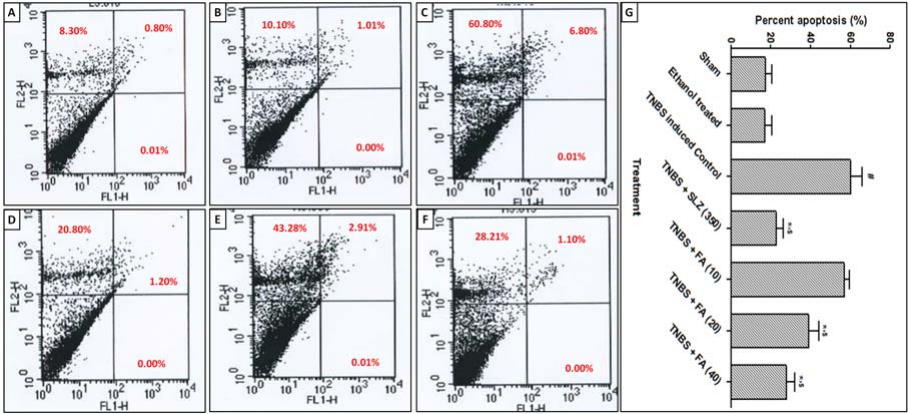
Effect of FA on TNBS-induced alteration in percent apoptosis (A-F). A representative image of apoptotic cell populations from sham group (A), ethanol-treated control group (B), TNBS-induced control group (C), TNBS-induced and Sulfasalazine (350 mg/kg, p.o.) treated group (D), TNBS-induced and FA (20 mg/kg, p.o.) treated group (E) and TNBS-induced and FA (40 mg/kg, p.o.) treated group (F) observed after FACS analysis using Annexin V/FITCPI stain. Four distinct cell distribution patterns are visible: normal viable cells (lower left quadrant), early apoptotic cells (lower right quadrant), necrotic cells (upper right quadrant and upper left quadrant). A quantitative representation of FA effect on TNBS-induced alteration in percent apoptosis (G). Data are expressed as mean ± SEM (n = 4) and analyzed by one-way ANOVA followed by Tukey's multiple range test. *p < 0.05 as compared to the TNBS-induced control group, ^#^p < 0.05 as compared to sham as well as ethanol-treated control group and ^$^p < 0.05 as compared to one another. SLZ (350): TNBS-induced and Sulfasalazine (350 mg/kg, p.o.) treated group; FA (10): TNBS-induced and Ferulic acid (10 mg/kg, p.o.) treated group; FA (20): TNBS-induced and Ferulic acid (20 mg/kg, p.o.) treated group and FA (40): TNBS-induced and Ferulic acid (40 mg/kg, p.o.) treated group.

There was a significant increase (*p* < 0.05) in the colonic apoptosis of TNBS-induced control rats as compared to sham as well as ethanol treated rats. When compared to TNBS-induced control rats, treatment with FA (20 and 40 mg/kg) significantly decreased (*p* < 0.05) the colonic apoptosis. FA (10 mg/kg) treatment showed non-significant down-regulation in colonic apoptosis as compared to TNBS-induced control rats. However, sulfasalazine (350 mg/kg) treatment significantly decreased (*p* < 0.05) the colonic apoptosis when compared to TNBS-induced control rats. Furthermore, decreased in colonic apoptosis was more significant (*p* < 0.05) in sulfasalazine (350 mg/kg) treated rats as compared to FA-treated rats (Figure 2(Fig. 2)).

### Effect of FA on histological alteration 

**Figure 3:**
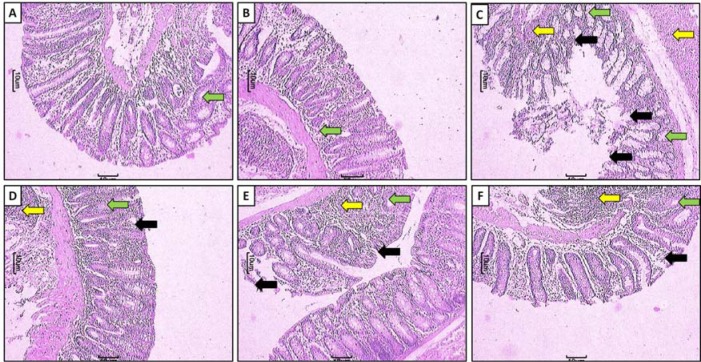
Effect of FA on TNBS-induced alteration in colon histology of rats. Photomicrograph of sections of colon of normal group (A), sham control group (B), TNBS-induced control group (C), TNBS-induced and Sulfasalazine (350 mg/kg, p.o.) treated group (D), TNBS-induced and FA (20 mg/kg, p.o.) treated group (E) and TNBS-induced and FA (40 mg/kg, p.o.) treated group (F). Images of H&E stain at 40X. Ulceration (black arrow), inflammatory infiltration (yellow arrow) and edema (green arrow).

Figure 3A(Fig. 3) and Figure 3B(Fig. 3) depicted the normal architecture of colon tissue from sham as well as ethanol treated rat which is devoid of any inflammatory infiltration necrosis and edema. Intra-rectal instillation of TNBS caused significant elevation (*p* < 0.05) in inflammatory response characterized by thickening of the mucosa, extensive destruction of mucosal epithelium, inflammatory cell infiltration with necrotic foci, submucosal edema, loss of mucus-secreting cells, necrosis and ulcer on the mucosal surface (figure 3C(Fig. 3)). Rats treated with FA (20 and 40 mg/kg) showed significant inhibition (*p* < 0.05) in TNBS-induced colonic histological damage which was characterized by significant reduction in the inflammatory infiltrate as well as mucosal thickening. FA showed the mild depletion of goblet cell. FA also showed the significant reduction (*p* < 0.05) of mucosal epithelium destruction, necrosis, ulceration and submucosal edema thus re-epithelialization of surface area (Figure 3E(Fig. 3) and Figure 3F(Fig. 3)). The section of colon from sulfasalazine (350 mg/ kg) treated rats showed a significant decrease (*p* < 0.05) in inflammatory infiltration, edema, and ulceration (Figure 3D(Fig. 3) and Table 3(Tab. 3)).

## Discussion

Ulcerative colitis is an inflammatory bowel disease that represents clinicopathologic features of inflammation of colonic mucosa. Several lines of evidence suggested evidence that imbalance in the mucosal immune system caused generation of inflammatory mediators such as reactive oxygen species (ROS) and cytokines (TNF, IL-1Β, and IL-6) which in turn leads to chronic inflammation, ulceration and lesions of the colonic mucosa, and morphologically represented as leukocyte infiltration, edema, and tissue injury (Kandhare et al., 2012[35]). TNBS-induced experimental colitis is a simple, reliable, reproducible, robust model widely used for screening of potential drug candidates for IBD (de Faria et al., 2012[14]; Kandhare et al., 2011[36]; Randhawa et al., 2014[54]). Intracolonic administration of an inflammatory or haptenizing agent like TNBS caused delayed-type hypersensitivity resulted in IL-12-mediated Th1 T cell transmural colitis which permits examination of effects of various agents with potent immune-inflammatory inhibitory activity (Randhawa et al., 2014[54]). In the present study, we have investigated the ameliorative effect of FA in the rat model of TNBS-induced colitis by assessing various biochemicals, molecular and histological changes. 

In the past, researchers have underlined the correlation between the pathogenesis of ulcerative colitis with oxidative stress in the both preclinical (Keshavarzian et al., 1990[41]) as well as clinical settings (D'Odorico et al., 2001[16]). It has been reported that activated phagocytic leukocytes have an ability to produce superoxide radicals that eventually leads to the production of reactive hydroxyl radical and peroxide (Adil et al., 2014[1]; Aswar et al., 2015[3]). Administration of TNBS may disrupt the endogenous antioxidant defense system leading to colonic oxidative damage (Li et al., 2011[47]). Superoxide dismutase (SOD) is an endogenous enzyme, whereas glutathione (GSH), a non-enzymatic antioxidant and both together play a vital role in cellular defense against elevated H_2_O_2_ generation (Kandhare et al., 2015[29]). Furthermore, malondialdehyde (MDA) is a highly toxic molecule which is a secondary product of lipid peroxidation. Elevated MDA level is a reliable and sensitive indicator of the severity and extent of increased lipid peroxidation in inflamed colon tissue. In the present study, decreased SOD and GSH levels were found in the colonic mucosa of colitis-induced rats which might be due to excessive free radical production. Treatment with FA significantly increased the SOD and GSH contents as well as decreased the MDA contents in TNBS-induced colitis rats. It may be due to the antioxidant potential of FA. Other studies also documented this antioxidant potential of FA to ameliorate ROS-induced damage in various disease states (Barros et al., 2008[5]; Ghaisas et al., 2014[18]).

Myeloperoxidase (MPO) is an enzymatic catalyst stored in azurophilic granules of polymorphonuclear neutrophils and macrophages. An elevated level of MPO considered as an indicator of neutrophil activation under the influence of pro-oxidative and pro-inflammatory conditions (Honmore et al., 2015[24]; Kandhare et al., 2012[34]). Similarly, we also found significantly elevated MPO levels in TNBS-induced colitis rats. Under stressful conditions, MPO catalyzes the formation of cytotoxic oxidants like hypochlorous acid from H_2_O_2_ and chloride ions (Yamada and Grisham, 1991[64]). Reduction in the activity of MPO enzyme after FA administration may be due to its anti-inflammatory potential. This notion is further supported by the histological finding of colonic tissue of FA treated rats where it effectively reduced the histological signs of inflammatory infiltration, edema, and tissue injury. The anti-inflammatory effects of FA via inhibition of MPO have been previously described (Islam et al., 2008[27]). Thus, findings of present investigation where FA suppressed the severity of TNBS colitis via inhibition of MPO activity is in line with the result reviewed elsewhere (Islam et al., 2008[27]).

It has been well documented that L-arginine is a precursor for the synthesis of nitric oxide (NO) via nitric oxide synthase (NOs) (Kandhare et al., 2015[32]). Expression of inducible NOs increases under oxidative stress conditions (Raygude et al., 2012[55]). NO in association with superoxide anion forms a poisonous peroxynitrite (ONOO^−^) anion which activates the nitrosative stress and caused dysregulation of inflammatory cells function that impairs the colonic mucosa (Dijkstra et al., 1998[15]). Interestingly, in the present study, we found a significantly elevated colonic mucosal NO activity with upregulated expression of iNOS. Administration of FA resulted in significant down-regulation of iNOS expression with a subsequent decrease in colonic NO level which seems to ameliorate the inflammatory response and tissue injury in experimental colitis. 

Hydroxyproline is an amino acid, and its quantification act as an important indicator for collagen synthesis (Kandhare et al., 2015[29]). In the present study, elevated hydroxyproline level was found after TNBS administration, and this increased hydroxyproline level significantly correlated with the accumulated collagen in colonic tissue. The results of the present investigation are in line with findings of the previous study (Motawi et al., 2012[49]). However, FA treatment significantly reduced the colonic hydroxyproline activity in TNBS-induced colitis rats which reflected tissue healing.

Immune cells hyperactivation plays a crucial role in the progression of IBD via the release of pro-inflammatory cytokines like TNF-α and IL-1β (Rojas-Cartagena et al., 2005[57]). These elevated levels of pro-inflammatory cytokines are observed not only in the inflamed gut of IBD patients but also in animals with TNBS-induced colitis (Hyam et al., 2013[26]). Increased TNF-α levels cause disruption of the epithelial barrier and induces the apoptosis of epithelial cells which leads to secretions of chemokines from intestinal epithelial cells (Bischoff et al., 1999[10]). IL-1β and IL-6 are important for gastrointestinal host defense however, their overproduction may lead to excessive gut inflammation and intestinal motility disorders (Bossone et al., 2001[11]). IL-6 is involved in recruitment and activation of neutrophils, which are believed to be important in exacerbations of IBD (Hyam et al., 2013[26]). It has been suggested that the infiltration of neutrophils into the mucosa significantly contributes to tissue necrosis and dysfunction (Grisham et al., 2002[19]). In addition, chemical moieties such as TNF-α and IL's antagonist was found to suppress the infiltration of inflammatory cells in the colonic tissue in an animal model of experimental colitis (Tountas et al., 1999[59]). In the current study, TNBS significantly elevated colonic mRNA expression of TNF-α, IL-1β, and IL-6 which may advocates enhanced immune inflammatory response. FA administration significantly attenuated the upregulated TNF-α, IL-1β, and IL-6 colonic mRNA expression. This immunomodulatory effect of FA might be beneficial against intestinal inflammation.

IL-10 is an anti-inflammatory cytokine and inhibits the production of pro-inflammatory cytokines and chemokines including TNF-α, IL-1β, and IL-6 (Moore et al., 2001[48]). It has been reported that IL-10 receptor-2 deficient mice exhibit intestinal inflammation mediated via T helper (Th)-1 response (Berg et al., 1996[8]) which reflects the important role of IL-10 in the regulation of cytokine expression in the mucosal immune system. Previous research has shown that TNBS-induced colitis is associated with decreased level of IL-10 in colon homogenates (Hyam et al., 2013[26]). Results of the present investigation are accordance with the findings previously study (Hyam et al., 2013[26]). Moreover, FA treatment significantly decreased the TNBS-induced down-regulation of intestinal IL-10 mRNA expression. This effect might be due to the anti-inflammatory activity of FA.

It has been well documented that Cyclooxygenase-2 (COX-2) is an inducible isoform of cyclooxygenase which serves as the rate-limiting enzyme and catalyzes the initial step of arachidonic acid metabolic transformation into prostanoids. Elevated levels of inducible enzyme COX-2 has been reported at the sites of inflammation during experimental colitis (de Faria et al., 2012[14]). Overproduction of TNF-α and nitric oxide (NO) association with COX-2 cause cellular degeneration in the inflamed colon (Kim et al., 2005[42]). Thus, elevated pro-inflammatory influx after TNBS administration caused elevated levels of COX-2 and iNOS, which was reversed by the FA treatment due to its anti-inflammatory potential. The results of present investigations are in line with the previous study, where FA inhibits protein expression of COX-2 and iNOS (Ojha et al., 2015[53]).

Apoptosis is a programmed cell death occurring in various inflammatory diseases including IBD (Wang et al., 2015[62]). It indicated a rapid switch from the inflammatory phase to the proliferative phase. In the present investigation, flow cytometric evaluation of colonic tissue from TNBS-induced control rats showed significantly increased apoptotic and necrotic cells in upper quadrants. The elevated apoptosis occurred due to rapid production and release of a pro-inflammatory cytokine such as TNF-α, which is responsible for tissue apoptosis (Kandhare et al., 2014[37]; Raygude et al., 2012[56]). Treatment with FA significantly reduced the cell apoptosis, as reflected by the decreased percentage of necrotic cells as compared to TNBS-induced control rats.

Recently, it has been shown that CAM therapy is used as an adjuvant therapy along with conventional therapy in 40 % of patients with IBD (Hilsden et al., 2011[22]). A randomized, double-blind, placebo-controlled study showed that wheatgrass juice (*Triticum aestivum*), aloe vera gel and *Andrographis paniculata* extract (HMPL-004) were superior to placebo (Ben-Arye et al., 2002[7]; Langmead et al., 2004[46]; Sandborn et al., 2013[58]). Furthermore, eighty-nine patients with UC randomized to curcumin (1 g twice daily) showed significantly lower relapse rate than the placebo group (Hanai et al., 2006[21]). Administration of *Boswellia serrata* (900 mg daily) showed an improvement in stool properties, histopathology, and scanning electron microscopy (Gupta et al., 2001[20]). *Plantago ovata* seeds also showed a promising effect in reduction and preventing clinical recurrence of postoperative IBD (Fernandez-Banares et al., 1999[17]).

It has been documented that the polyphenol compound that possesses one hydroxyl group in the aromatic ring seemed to have potent antioxidant activity (Nenadis et al., 2003[50]). Moreover, the presence of electron-donating groups attached to the aromatic ring such as -OCH3 and -OH ought to increase free radical scavenging potential. The presence of electron-donating -OH groups attached to the para or 4-position of the aromatic ring in FA owing to a greater number of canonical resonance that eventually contribute to its antioxidant property (Karamac et al., 2005[40]). Furthermore, previous molecular docking study of FA with Cyclooxygenase-2 showed that it forms dimer via the formation of a strong hydrogen bond between -OH of -COOH group of one FA molecule with the oxygen atom of the C = O group of adjacent molecule to serves as potent inhibitors of COX-2 enzyme (Kumar and Pruthi, 2015[44]).

Sulfasalazine (SLZ) is a sulfa drug currently used for the treatment of human IBD. It is poorly absorbed in the gut but, its metabolite 5-aminosalicylic acid has been effective against colitis (Horvath et al., 2008[25]). An array of evidence suggests that SLZ reduces inflammation by various mechanisms including inhibition of cyclooxygenase and 5-lipoxygenase pathway of arachidonic acid metabolism (Horvath et al., 2008[25]; Witaicenis et al., 2012[63]). In the present study, administration of SLZ (350 mg/kg) as well as FA (20 and 40 mg/kg) reduced the number of intestinal epithelial cell apoptotic damage via different mechanisms including inhibition of colonic oxidative stress, the release of pro-inflammatory mediators and COX-2. Thus, it is possible that the inhibitory effect of FA or SLZ on these mediators could be comparable, at least in part, to inhibit TNBS-induced colitis.

Thus, anti-inflammatory activity exhibited by FA in the TNBS-induced colitis can be attributed to its ability to inhibit oxido-nitrosative stress, apoptosis, proinflammatory cytokines production, and down-regulation of COX-2 synthesis.

## Acknowledgements

The authors would like to acknowledge Dr. S. S. Kadam, Vice-Chancellor and Dr. K. R. Mahadik, Principal, Poona College of Pharmacy, Bharati Vidyapeeth Deemed University, Pune and for providing necessary facilities to carry out the study. The authors also acknowledge Invaluesys Research Group to carry out statistical analysis of the study.

## Conflict of interest

The authors declare no conflict of interest.

## Supplementary Material

Supplement

## Figures and Tables

**Table 1:**
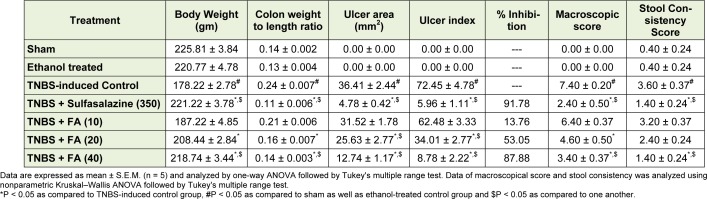
Effect of FA on TNBS-induced alterations in body weight, colon weight to length ratio, ulcer area, ulcer index, macroscopic score and stool consistency score in rats

**Table 2:**
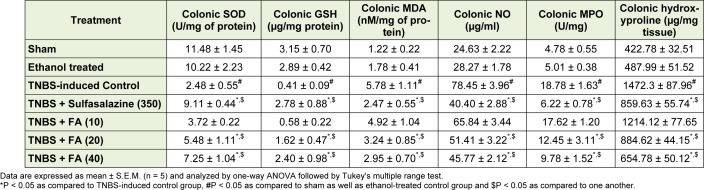
Effect of FA on TNBS-induced alterations in colonic SOD, GSH, MDA, NO, MPO and hydroxyproline activity in rats

**Table 3:**
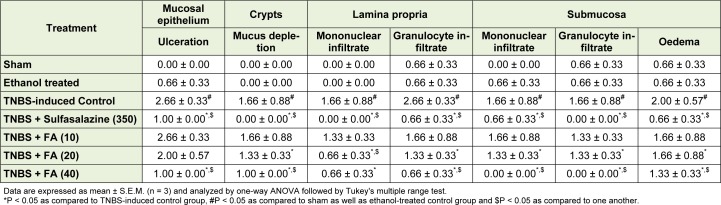
Effect of FA on TNBS-induced alteration in histology of rat colon
