# Supplementary material for: Ferulic acid ameliorates TNBS-induced ulcerative colitis through modulation of cytokines, oxidative stress, iNOs, COX-2, and apoptosis in laboratory rats
Source: EXCLI J. 2016 Aug 9;15:482–99. doi: 10.17179/excli2016-393 (PMC5083962; doi:10.17179/excli2016-393)
Supplement: Supplement [file EXCLI-15-482-s-001.pdf]

EXCLI Journal 2016;15:482-499 – ISSN 1611-2156
Received: May 17, 2016, accepted: July 07, 2016, published: August 09, 2016

Supplementary material to:

FERULIC ACID AMELIORATES TNBS-INDUCED ULCERATIVE COLITIS THROUGH MODULATION OF CYTOKINES, OXIDATIVE STRESS, INOS, COX-2, AND APOPTOSIS IN LABORATORY RATS

Smeeta S. Sadar[1], Niraj S. Vyawahare[1], Subhash L. Bodhankar[2*]

[1] Padmashree Dr. D. Y. Patil College of Pharmacy, Akurdi, Pune Maharashtra, 411044, India
[2] Department of Pharmacology, Poona College of Pharmacy, Bharati Vidyapeeth Deemed University, Pune, Maharashtra, 411038, India

* Corresponding author: Dr. S. L. Bodhankar, Dept. of Pharmacology, Poona College of Pharmacy, Bharati Vidyapeeth Deemed University, Erandwane, Pune-411038, Maharashtra, E-mail: drsbodh@gmail.com, smeeta.mohod@rediffmail.com

http://dx.doi.org/10.17179/excli2016-393

This is an Open Access article distributed under the terms of the Creative Commons Attribution License (http://creativecommons.org/licenses/by/4.0/).

EXCLI Journal 2016;15:482-499 – ISSN 1611-2156
Received: May 17, 2016, accepted: July 07, 2016, published: August 09, 2016

Supplementary File 1: TNBS induced-colitis and drug treatment schedule

Group No.	Animal group	Treatment	Dose (mg/kg)	Route of administration	No. of animals	Day of treatment 0	Day of treatment 1 to 14	Day of treatment 15
I	Sham	Dimethyl sulfoxide (DMSO) (1%)	1 ml	p. o.	10	---	√	Blood sample were collected and animals were sacrificed for bio-chemical, molecular and histo-pathological examination
		Physiological saline	0.25 mL	Intrarectally		√	---	
II	Ethanol treated	DMSO (1%)	1 ml	p. o.	10	---	√	
		Ethanol (50%)	0.25 mL	Intrarectally		√	---	
III	TNBS-induced control	DMSO (1%)	1 ml	p. o.	10	---	√	
		TNBS	100 mg/kg	Intrarectally		√	---	
IV	TNBS-induced and Sulfasalazine treated	Sulfasalazine	50 mg/kg	p. o.	10	---	√	
		TNBS	100 mg/kg	Intrarectally		√	---	
V	TNBS-induced and Ferulic acid (10 mg/kg) treated	Ferulic acid	10 mg/kg	p. o.	10	---	√	
		TNBS	100 mg/kg	Intrarectally		√	---	
VI	TNBS-induced and Ferulic acid (20 mg/kg) treated	Ferulic acid	20 mg/kg	p. o.	10	---	√	
		TNBS	100 mg/kg	Intrarectally		√	---	
VII	TNBS-induced and Ferulic acid (40 mg/kg) treated	Ferulic acid	40 mg/kg	p. o.	10	---	√	
		TNBS	100 mg/kg	Intrarectally		√	---	

Supplementary File 2: Primer sequences for TNF-α, IL-1β, Il-6, IL-10, COX-2, iNOs, and β-actin

Gene	Sequence: Forward primer	Sequence: Reverse primer	Size (bp)
**TNF-α**	AAGCCTGTAGCCCATGTTGT	CAGATAGATGGGCTCATACC	295
**IL-1β**	TGATGTTCCCATTAGACAGC	GAGGTGCTGATGTACCAGTT	290
**IL-6**	TAGCCGCCCCACACAGACAG	GGCTGGCATTTGTGGTTGGG	479
**IL-10**	AGA-AGG-ACC-AGC-TGG-ACA-AC	GTC-GCA-GCT-GTA-TCC-AGA-GG	204
**COX-2**	ACAACATTCCTTCCTTC	CCTTATTTCCTTTCACACC	253
**iNOs**	ATCCCGAAACGCTACACTT	TCTGGCGAAGAACAAATCC	314
**β-actin**	GTCACCCACACTGTGCCCATCT	ACAGAGTACTTGCGCTCAGGAG	764
